# Interface of Phospholipase Activity, Immune Cell Function, and Atherosclerosis

**DOI:** 10.3390/biom10101449

**Published:** 2020-10-15

**Authors:** Robert M. Schilke, Cassidy M. R. Blackburn, Temitayo T. Bamgbose, Matthew D. Woolard

**Affiliations:** Department of Microbiology and Immunology, Louisiana State University Health Sciences Center, Shreveport, LA 71130, USA; rschil@lsuhsc.edu (R.M.S.); cragai@lsuhsc.edu (C.M.R.B.); tbamgb@lsuhsc.edu (T.T.B.)

**Keywords:** atherosclerosis, phospholipases, macrophages, T cells, lipins

## Abstract

Phospholipases are a family of lipid-altering enzymes that can either reduce or increase bioactive lipid levels. Bioactive lipids elicit signaling responses, activate transcription factors, promote G-coupled-protein activity, and modulate membrane fluidity, which mediates cellular function. Phospholipases and the bioactive lipids they produce are important regulators of immune cell activity, dictating both pro-inflammatory and pro-resolving activity. During atherosclerosis, pro-inflammatory and pro-resolving activities govern atherosclerosis progression and regression, respectively. This review will look at the interface of phospholipase activity, immune cell function, and atherosclerosis.

## 1. Introduction

All cellular membranes are composed mostly of phospholipids. Phospholipids are amphiphilic compounds with a hydrophilic, negatively charged phosphate group head and two hydrophobic fatty acid tail residues [1]. The glycerophospholipids, phospholipids with glycerol backbones, are the largest group of phospholipids, which are classified by the modification of the head group [1]. The negatively charged phosphate head forms an ionic bond with an amino alcohol. This bridges the glycerol backbone to the nitrogenous functional group (amino alcohol). The addition of an amino alcohol largely dictates the quaternary structure of the phospholipid [2]. A smaller but also critical family of phospholipids are the sphingolipids, which have sphingosine as a backbone [3]. The amphiphilic make-up of phospholipids allows them to create lipid bilayers, which make cellular membranes and supply structure to cells. Phospholipids also contribute to cellular responses through the binding of receptors, such as lysophosphatidic acid (LPA) binding to the family of LPA receptors, and sphingosine-1-phosphate (S1P) binding to S1P receptors [4]. Components of phospholipids, such as inositol trisphosphate (IP3), diacylglycerol (DAG), and fatty acids, are substrates for the activation of intracellular receptors (e.g., inositol trisphosphate receptors), cofactors for proteins (e.g., protein kinase C), and transcription factors (e.g., peroxisome proliferator-activated receptors (PPARs) [5]. In addition, free fatty acids are also precursors to the prostanoid family of lipid mediators, which can have a broad array of cellular and physiological effects.

**Figure 1:**
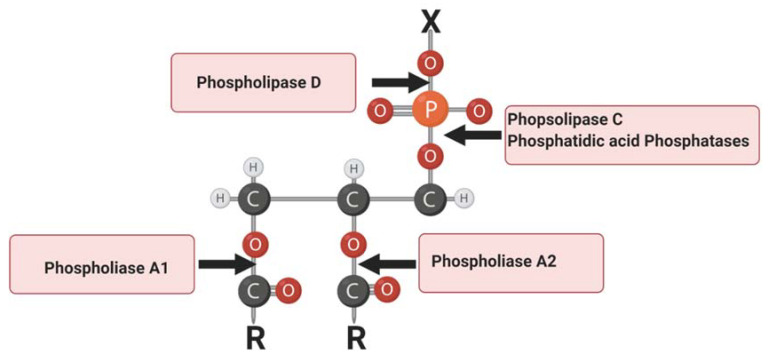
Schematic representation of phospholipase enzymatic sites on phospholipids. “X” represents a functional group. Red “O” represents oxygen; orange “P” represents phosphorus; grey “C” represents carbon; white “H” represents hydrogen; “R” represents fatty acid tails. Figure Created with BioRender.com.

Phospholipases are a group of enzymes that cleave phospholipids. Each family of phospholipases cleaves a unique site on a phospholipid or unique phospholipid family. Phospholipase A hydrolyzes the fatty acid esters from the sn-1 (PLA1) or sn-2 (PLA2) position of the glycerol backbones, generating free fatty acids [6]. Phospholipase C (PLC) hydrolyzes the glycerol linkage glycerophosphate bond of the polar head, generating DAG and IP3. Phospholipase D (PLD) hydrolyzes the head group of phospholipids, leaving phosphatide and phosphatidic acid (Figure 1). Phosphatidic acid phosphatases are a family of enzymes that can cleave phosphate heads from LPA, PA, and S1P (Figure 1). Phosphatidic acid phosphatases can be split into two families of enzymes, the LPPs, which cleave phosphate heads of lipids on the external side of the plasma membrane, and lipins, which cleave PA intracellularly. Phospholipases are critical regulators of the liberation of bioactive compounds contained within phospholipids and subsequent physiological activity of those compounds.

Atherosclerosis is an immuno-metabolic disease that leads to myocardial infarction, stroke, or sudden death [7]. Excess circulating cholesterol in the form of low-density lipoproteins (LDLs) can be deposited into the arterial intima. If these LDLs are not quickly removed, they can be modified (e.g., to oxidized LDL (oxLDL)) through a variety of enzymatic and nonenzymatic modifications [8]. Oxidative stress is associated with the increased oxidation of LDL and increased atherosclerosis progression. Oxidative stress occurs when there is an imbalance in the ratio of reactive oxygen species (ROS) and antioxidants [9,10]. ROS oxidize the polyunsaturated fatty acids and apolipoprotein B-100 on the LDL [11,12]. Once formed, oxLDL leads to the recruitment and activation of inflammatory cells into the arterial intima. Monocytes/macrophages are the main cells recruited into the intimal space to clear both LDL and oxidized LDL. Macrophages engulf LDL (non-oxidized) via the low-density lipoprotein receptor (LDLR). This initiates a negative feedback inhibitory loop resulting in the downregulation and degradation of the LDLR. The negative feedback loop reduces LDL uptake to limit intracellular cholesterol. However, after oxidation, macrophage scavenger receptors, such as CD36, increase oxLDL engulfment, leading to intracellular cholesterol accumulation and lipid droplet formation.

**Figure 2:**
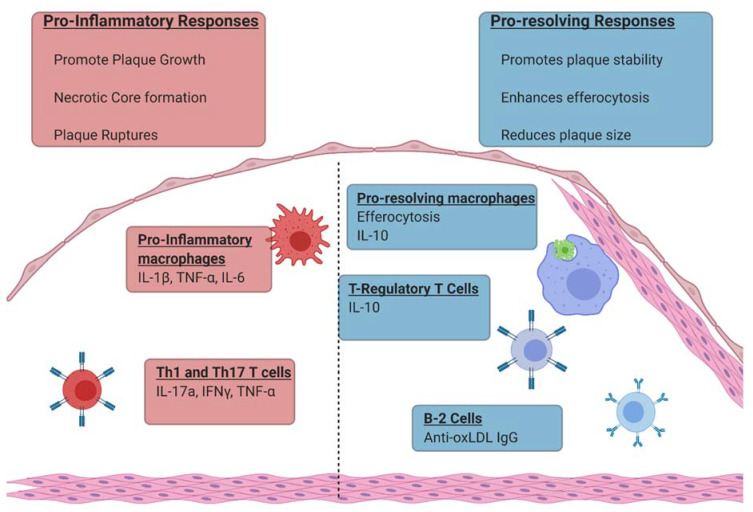
Immunological responses that contribute to plaque progression and plaque regression. Figure Created with BioRender.com.

A broad range of immune cells and immunological mediators contribute to atherosclerosis. Macrophages have long been recognized as a key component of the immune response that determine atherosclerosis severity [13]. It is now well established that neutrophils, dendritic cells, T cells, and B cells have important cellular responses within atherosclerotic plaque lesions as well [14]. Furthermore, immune mediators such as pro-inflammatory cytokines (IL-1β, TNF-α, and IL-6), anti-inflammatory cytokines (IL-10), and lipid mediators such as prostaglandins and pro-resolvins also contribute. Pro-inflammatory macrophage responses, Th1 and Th17 T cell responses, and the cytokines and prostaglandins those cells produce promote atherosclerotic progression (Figure 2) [15,16]. By contrast, pro-resolvins, macrophage efferocytosis, anti-phospholipid B cell responses, and T regulatory cell (Treg) responses promote atherosclerosis regression (Figure 2). Phospholipase activity has been documented to contribute to both pro-inflammatory and pro-resolving immune responses as well [17]. This review will concentrate on the contribution of phospholipases to atherosclerosis within immune responses.

## 2. Lipoprotein-Associated Phospholipase A2

Lipoprotein-associated phospholipase A2 (Lp-PLA2) is a 45 kDa monomeric protein and belongs to the phospholipase A2 superfamily [18]. Lp-PLA2 differs from the other phospholipase A2 members, as it does not require calcium for its enzymatic activity [13], and in its substrate specificity, as it preferentially hydrolyzes the oxidatively truncated sn-2 acyl chain of water-soluble phospholipids [19]. The enzyme is also known as platelet-activating factor acetyl hydrolase, due to its ability to hydrolyze and inactivate platelet-activating factor (PAF) [20]. Lp-PLA2 was initially suggested to play an atheroprotective role due to its enzymatic activity of hydrolyzing oxidized phospholipids in LDL and its function in degrading pro-inflammatory and atherogenesis-inducing PAF [21,22,23,24]. However, there are controversies regarding the effects of Lp-PLA2 on atherosclerosis [25].

Lp-PLA2 is encoded by the *PLA2G27* gene, which contains 12 exons. The *PLA2G27* gene is characterized by a variety of nonsynonymous polymorphisms that either attenuate Lp-PLA2 enzymatic activity or result in its complete loss [26]. Loss-of-function Lp-PLA2 is associated with an increase in cardiovascular disease, suggesting an atheroprotective role for the enzyme [22,27,28]. The loss of Lp-PLA2 activity is speculated to increase circulating PAF levels and increase the amounts of oxLDL. Lp-PLA2’s proposed atheroprotective role is also attributed to the predominant association of Lp-PLA2 with high-density lipoprotein (HDL) in mice [22]. However, LDL is low in mouse species compared to humans, suggesting a potential discrepancy for the contribution of Lp-PLA2 during atherosclerosis in humans [29]. Currently, Lp-PLA2 is considered a marker of cardiovascular disease. To further support this, Singh et al. reported an increase in the number of atherosclerotic lesions in transgenic mouse models that had greater amounts of Lp-PLA2 associated with LDL [30]. However, rather than it playing an atherogenic role, it is speculated that the correlation of atherosclerosis with increased amounts of Lp-PLA2 is a result of the protective function of the enzyme [25].

Lp-PLA2 is secreted by a variety of white blood cells and other specialized cells such as hepatocytes and adipocytes [31]. Lp-PLA2 synthesis and release into the circulation have been found to predominantly occur during monocyte maturation into macrophages [32]. In humans, circulating Lp-PLA2 is bound to lipoproteins, with 70–80% of the enzyme bound to apolipoprotein B on LDL, while the remaining is carried on HDL [33]. Specific residues in the Lp-PLA2 N-terminus bind the electronegative domain of apolipoprotein B (ApoB) on the C-terminus of LDL [34]. The Lp-PLA2 association with ApoB is increased as ApoB becomes more negatively charged [34]. While Lp-PLA2 associates with LDL in the blood, its potential atherogenic activity is not observed until it is found within the arterial intima [35]. Within the arterial intima, LDLs can be oxidized, providing the oxidatively truncated sn-2 chains that Lp-PLA2 is preferentially known to hydrolyze on phospholipids [36]. Lp-PLA2 mediates the hydrolysis of oxidized LDL, yielding oxidized non-esterified fatty acids (oxNEFA) and lysophosphatidylcholine (LysoPC) [35]. These two hydrolytic products are individually and collectively pro-inflammatory and atherogenic [36]. oxNEFA and LysoPC induce the apoptosis of macrophages and increase the recruitment of leukocytes in the sub-intimal space of the artery wall [35,37]. This eventually facilitates the development of the plaque lipid core [26].

LysoPC, in particular, encompasses multiple atherogenic and pro-inflammatory activities because it acts as a monocyte chemoattractant factor, induces oxidative stress, induces endothelial dysfunction, upregulates the expression of adhesion molecules and cytokines (IL-1β, IL-6, and TNF-α), and induces apoptosis in endothelial cells, smooth muscle cells, and macrophages [35,37,38]. Increased amounts of LysoPC were found in patients with early coronary atherosclerosis when compared with control subjects [39]. Conversely, it is speculated that LysoPC does not pose much of an atherogenic threat because LysoPC is mostly found in a bound state, thus reducing its availability [25]. Consequently, the amount of LysoPC measured in the plasma is not a true representation of the amount of LysoPC that is biologically available.

Apoptotic cells are phagocytosed by neighboring macrophages in a receptor–ligand interaction called efferocytosis. Defects in efferocytosis are one of the biggest drivers of atherosclerotic plaque growth and the formation of necrotic cores that lead to destabilized plaques. The macrophage scavenger receptor CD36 recognizes exposed oxidized phosphatidylcholine and phosphatidylserine molecules on the surface of apoptotic cells. The Lp-PLA2 cleavage of oxidized phosphatidylcholine reduces the scavenger receptor recognition of apoptotic cells by macrophages [40]. The impaired clearance of apoptotic cells leads to necrosis and the subsequent expansion of the necrotic core [41]. The Lp-PLA2-induced formation of oxNEFA can also elicit monocyte and leukocyte recruitment and induce apoptosis [35,37]. The combination of enhanced leukocyte recruitment, increased apoptosis, and reduced efferocytosis are likely responsible for the expansion of the necrotic core and the thinning of the fibrous cap [35,42].

Lp-PLA2 mRNA has not only been found to be upregulated in atherosclerotic plaques but has also been shown to be strongly expressed in the macrophage populations that are found within the fibrous cap of vulnerable atherosclerotic plaques [43,44]. The presence of Lp-PLA2 substrates and products of its hydrolytic activity in lipid-laden plaques further supports the atherogenic role of Lp-PLA2 [45]. An autopsy examination study on 25 sudden coronary death patients found Lp-PLA2 to be highly upregulated in the ruptured plaques found in the human coronary arteries and their cap fibroatheromas [46]. Several large studies have continued to show that Lp-PLA2 is an independent and reliable predictor of cardiovascular diseases [47,48]. Based on these pieces of evidence and the recommendations of several major international societies, Lp-PLA2 is considered a cardiovascular disease risk factor by the Food and Drug Administration [49]. In summary, the enzymatic activity of Lp-PLA2 and the products of its hydrolytic action facilitate the continuous progression and detrimental destabilization of atherosclerotic plaques.

## 3. Lipid Phosphate Phosphatases

Lipid phosphate phosphatases (LPPs) are a group of enzymes that belong to the phosphatase/phosphotransferase family. LPPs dephosphorylate phosphatidic acid, lysophosphatidic acid (LPA), sphingosine-1-phosphate (S1P), ceramine-1-phosphate (C1P), and diacylglycerol pyrophosphate [50]. LPPs are typically localized on the plasma membranes, with the outer leaf containing the active site. LPPs can also be expressed on the membranes of the endoplasmic reticulum (ER) and Golgi, allowing the metabolism of internal lipid phosphates [51]. LPPs modify the concentrations of lipid phosphates and their dephosphorylated products to regulate cell signaling [52]. LPPs regulate cell signaling through the dephosphorylation of bioactive lipids. As mentioned above, LPPs dephosphorylate lipid products such as LPA, S1P, and C1P. LPA activates PPARs and nuclear LPA1 receptors, resulting in an increase in transcription and cell signaling pathways such as those involved in cell proliferation, migration, calcium mobilization, etc. [53,54,55]. S1P elicits calcium mobilization, ERK activity, and protection against apoptosis [56,57,58]. C1P promotes cell division and prevents apoptosis. The LPP-mediated degradation of LPA, S1P, and C1P will terminate the receptor-mediated activities.

LPPs have three isoforms—LPP1, LPP2, and LPP3—that each have a conserved catalytic domain to dephosphorylate lipid phosphates [51,59]. LPP3 also has noncatalytic activity that allows it to bind to integrins. This noncatalytic activity promotes endothelial cell-to-cell adhesion and depends on the arginine–glycine–aspartate recognition motif [60,61]. Each LPP contributes to different cell responses in various models of inflammation. For example, ovarian cancer cells are exposed to an elevated amount of LPA, which results in cell proliferation and survival. Ovarian cancer cells also have reduced LPP1 mRNA [62]. When LPP1 is overexpressed in ovarian cancer cells, LPA hydrolysis is increased and results in decreased cell proliferation and increased apoptosis [62]. Within platelets, LPP1 dephosphorylates LPA, which may help to recruit monocytes and macrophages after endothelial cell and vascular muscle cell stimulation [63]. Increased plasma LPA may also participate in signaling and stimulation for the growth of tumor cells and is associated with increased gynecological cancers [63]. The inducible inactivation of the LPP3 gene in endothelial and hematopoietic cells enhanced inflammation in mice after challenge with LPS or thioglycolate [64]. LPP3 overexpression in HEK293 cells increases phosphatidic acid-to-diacylglycerol conversion [51,65,66]. Altered phosphatidic acid/diacylglycerol concentrations affect different cellular processes. For example, within neutrophils, membrane-associated phosphatidic acid stimulates endothelial cell tyrosine kinases, which results in increased membrane permeability in the endothelial cells. LPP activity reduces membrane-associated phosphatidic acid and therefore stifles endothelial cell membrane permeability [64]. Overall, LPPs are involved in numerous different cell processes and are regulated by lipid phosphate availability to influence cell cycle and inflammatory responses.

Single nucleotide polymorphisms have been identified in *PLPP3* (the gene that encodes LPP3) that are associated with an increased risk of coronary artery disease [67,68,69]. LPP3 can be detected in human atheromas and is mainly found in foam cells [70]. Further investigation showed oxidized LDL upregulates the *PLPP3* gene and associated LPP3 protein expression within macrophages [70]. Specifically, oxidized LDL increases the enzymatic activity of LPP3. The atheroprotective role of LPP3 may be through the reduction of LPA. LPA increases plaque-associated thrombosis [71]. Multiple animal models of atherosclerosis have shown LPP3 is upregulated in endothelial cells, CD68-positive cells (monocytes/macrophages), and smooth muscle cells [68]. In mice, LPP3 is necessary during early vascular development; global deletion causes embryonic lethality [64,72]. Mice with an induced global deletion of *PLPP3* have larger atherosclerotic plaques associated with increased lesional LPA [68]. Liver-specific, conditional *PLPP3* knockout mice crossed with apolipoprotein E (ApoE) knockout mice have significantly larger plaques and necrotic cores within aortic roots compared to wild-type ApoE knockout mice. The authors show that the deletion of liver-specific LPP3 increased atherogenic lipids, such as LPA and other lysophosphatidylinositols, in the plasma [73]. The increase in atherogenic lipids correlated with increased atherosclerosis progression [73].

Oxidized LDL-treated bone marrow-derived macrophages have increased LPP3 expression, suggesting macrophage LPP3 may regulate atherosclerosis progression. However, in a model of atherosclerosis, myeloid-derived *PLPP3* does not increase LPA lesion localization or increase atherosclerosis progression. Along with macrophages, smooth muscle cells are also able to transition into foam cells during atherosclerosis. The deletion of smooth muscle cell LPP3 resulted in increased atherosclerosis plaque growth [68]. The authors demonstrated that LPP3-deficient smooth muscle cells still transition to foam cells but may have altered responses to lipids that lead to increased plaque growth and inflammation. These data suggest smooth muscle cell LPP3 is atheroprotective. The above studies demonstrate that LPP3 is involved in atherosclerosis. More work is needed to truly understand the cell-specific contributions of LPP3 and the contributions of LPP1 and LPP2 toward atherosclerosis.

## 4. Phospholipase C

Phospholipase C (PLC) is a calcium-dependent phosphodiesterase that regulates phosphoinositide metabolism. PLC hydrolyzes phosphatidylinositol 4,5-bis-phosphate (PI(4,5)P_2_) to generate the second messengers inositol 1,4,5-trisphosphate (IP_3_), and diacylglycerol (DAG) [74]. There are thirteen PLC isozymes in mammals, which are categorized into six classes based on structure. These classes include PLC β, γ, δ, ϵ, λ, and ν [74]. These structures largely dictate interactions with cell surface receptors including G-protein-coupled receptors (GPCRs), G-proteins, receptor tyrosine kinases (RTKs), and non-receptor tyrosine kinases [74]. There are numerous reviews focusing on the structure and regulation of each class of PLCs [75,76,77]; as such, those will not be covered here. Rather, we will review what is known about PLC and its contribution to atherosclerosis and immune responses.

Phospholipase C is known to regulate multiple immunological responses of T and B lymphocytes [78]. T cell receptor signaling results in the activation of PLC. The PLC-mediated cleavage of PI(4,5)P_2_ generates IP_3_ and DAG, which both have significant roles in the activation of immune cells. DAG activates protein kinase C (PKC), resulting in the initiation of NFκβ signaling to promote inflammatory gene transcription [79,80]. IP3 binds to the IP3 receptor, leading to calcium release from the endoplasmic reticulum. Calcium activates calcineurin, resulting in the nuclear translocation of NFAT to promote IL-2 production and subsequent T cell proliferation [81]. In addition, PLC deficiency leads to a reduction of Treg development, which may promote chronic inflammation [82]. PLC plays a similar role in B cell activation as it does in T cells by promoting downstream NFκB- and NFAT-mediated transcription. This is accomplished through IP_3_- and DAG-mediated signaling [80].

In comparison to those in lymphocytes, the functional consequences of PLC-mediated signaling in myeloid cells are diverse. PLC is required for macrophage differentiation in response to macrophage colony-stimulating factor (MCSF) [78,83]. In addition to promoting differentiation, activated macrophages and dendritic cells require PLC for appropriate cytokine production and dendritic cell migration [78,84]. Upon entry into tissue, macrophages and dendritic cells constitutively engulf surrounding antigens and present them on the cell surface. This engulfment requires the synthesis of phosphatidic acid (PA), and PLC is required for the generation of intermediates of the PA synthetic pathway, leading to subsequent RAC activation and actin polymerization [85]. PLC localizes to nascent phagosomes to promote the recruitment of PKC, leading to the uptake of IgG-opsonized antigens [85]. There are numerous studies demonstrating the critical role of PLC in immune cell activation and differentiation.

Although not extensively studied, the diverse role of PLC in immunological cells would suggest that phospholipase C likely contributes to the development of atherosclerosis. As previously mentioned, atherosclerosis is a chronic inflammatory disease, and PLC contributes to the activation and development of immune cells. Monocyte infiltration and reduced macrophage clearance exacerbate atherosclerosis [86]. PLC regulates the migration and phagocytic capacity of macrophages [78,84]. PLCβ3/ApoE-deficient mice exhibited a reduction in atherosclerotic lesion size in the aortic vessels, arches, and roots compared with littermate controls [87]. PLCβ3 deficiency also resulted in a reduction in the number of macrophages within murine atherosclerotic plaques [87]. The products of PLC enzymatic activity stimulate PKC, which is known to be atherogenic. PKC α/β positively regulates foam cell formation, and the deletion of PKCβ from ApoE KO mice reduced atherosclerotic plaque size [88,89]. Investigating the contribution of PLC within immune cells in atherosclerosis needs to be further explored.

Given that atherosclerosis is a chronic inflammatory condition, adaptive immune responses play a critical role in the progression of the disease. Immune responses from recruited T cells and B cells become the dominant factors that enhance local inflammation. Inflammatory T cell subsets (Th1) promote continued inflammation, which further exacerbates atherosclerosis. The inhibition of Th1 differentiation and cytokine production reduced the plaque area in the aortic root of atherosclerotic mice [90]. The inhibition of Th1 responses resulted in an increase in Th2 T cells, which led to a decrease in plaque area. B cell responses are largely atheroprotective, due to the production of immunoglobulins [91]. In particular, IgM and IgG directed at the epitopes of oxLDL seem to neutralize the pro-inflammatory epitopes [91]. Overall, the role of PLC in regulating T and B cell activation and function could have drastic impacts on atherosclerosis progression.

## 5. Phospholipase D

Phospholipase D (PLD) is a phospholipid-specific phosphodiesterase in which the enzymatic activity cleaves phosphorylcholine into phosphatidic acid and free choline [92]. PLD’s enzymatic activity has pleiotropic effects on a variety of cellular pathways. Mammalian phospholipase Ds are divided into two classical isoforms, PLD1 and PLD2, which have both redundant and specific functions depending on the tissue distribution [92].

Phospholipase D is regulated transcriptionally and post-translationally. Both PLD1 and PLD2 are activated by the presence of phosphatidylinositol 4,5-bisphosphate (PtdIns(4,5)P2) [92,93]. Other lipid species also activate PLD, such as PtdIns(3,4,5)P3 and unsaturated fatty acids [92,94,95]. Not only do lipid species regulate PLD, but proteins that regulate the abundance, location, and phosphorylation state of Ptdlns(4,5)P2 are also involved in the regulation of PLD [93,94]. Various stimuli, such as PDGF, EGF, or IL-1β, result in the increased gene expression of PLD via the activation of NFκB [96]. PLD is post-translationally modified by phosphorylation and palmitoylation. Phosphorylation by GTPases, such as ARF and Rho family proteins, directly activates PLD enzymatic activity [94,97,98]. Palmitoylation has been shown to alter the localization of PLD within the cell, from perinuclear to plasma membrane regions [99,100]. This shows the highly dynamic nature of phospholipase D within the cell.

Understanding how PLD contributes to chronic inflammatory diseases, such as atherosclerosis, may have significant implications in disease progression. PLD has been shown to be present within macrophages of a human atherosclerotic plaque [101]. PLD regulates phagocytosis in macrophages through the generation of phosphatidic acid. PLD1 vesicles are recruited to both nascent and internalized phagosomes, while PLD2 is observed at nascent phagosomes [101]. The shRNA depletion of either PLD1 or PLD2 results in a reduction in the phagocytic capabilities for IgG-coated latex beads of RAW264.7 macrophages [102]. Ganesan et al. investigated the role of PLD in the phagocytosis of oxidized LDL. They show that PLD2 is critical for the uptake up of oxidized LDL through the regulation of WASP and Grb2 to polymerize actin at the phagocytic cup [103]. PLD2 is also needed for the CD36-mediated removal of aggregated oxLDL [103]. Given the importance of lipid metabolism in immunological cells, PLD activity presumably plays a greater role in the progression of atherosclerosis than the current literature suggests. Neutrophil responses are known to promote early atherogenesis. In neutrophils, FcgammaR1 binding leads to PLD activation, which is critical for the oxidative burst during degranulation [104]. PLD recruits cytochrome B to the mitochondria to increase NADPH oxidase activity and ROS generation [105]. In addition, PLD indirectly activates the p22phox subunit of cytochrome D via PA production [106]. The PLD-mediated activation of neutrophils may promote early plaque progression. Altogether, phospholipase D is critical for various immunological responses, and the contribution of PLD to atherosclerosis needs to be further investigated.

## 6. Cytosolic Phospholipase A2

Cytosolic phospholipase A2 (cPLA2) is one of three categories of phospholipase A2s. The other phospholipase A2s are known as secretory PLA2 and calcium-independent PLA2 [107]. Phospholipase A2s catalyze the hydrolysis of glycerophospholipids to produce arachidonic acid metabolites [107]. Of the phospholipases, cPLA2 is highly selective for arachidonic acid-containing glycerophospholipids [107]. cPLA2 is a ubiquitous enzyme that is found in most tissues and cells; however, mature T and B lymphocytes do not have any detectable levels of cPLA2 [108,109]. There are three isoforms of cPLA2: cPLA2 beta (110 kDA), cPLA2 gamma (60 kDA), and cPLA2 alpha (85 kDA). Each isoform has two catalytic domains: A and B. Catalytic domain A contains the lipase consensus sequence GXSGS [109]. Inactive cPLA2 exists in the cytosol; however, upon calcium binding to the C2 domain, cPLA2 translocates to the endoplasmic reticulum (ER), Golgi apparatus, and nuclear envelope [107]. Steady intracellular calcium greater than 100–125 nM causes cPLA2 translocation to the Golgi, whereas steady intracellular calcium greater than 210–280 nM causes cPLA2 translocation to the Golgi, ER, and nuclear envelope [109]. cPLA2 cellular localization can have effects on different lipid-mediated processes. For example, a study with renal cells demonstrated cPLA2 localization at the Golgi can change the lipid ratio and result in changes in structure and protein trafficking [110]. Along with intracellular calcium levels, the phosphorylation of cPLA2 at Ser 505, Ser 515, and Ser 727 regulates cPLA2 activity [107]. Mitogen-activated protein kinase phosphorylates the above serine residues; phosphorylation increases the enzymatic activity [107,111]. The activation of cPLA2 leads to the liberation of arachidonic acid, which can be converted into inflammatory eicosanoids including prostaglandins.

cPLA2 activity promotes pro-inflammatory immune cell activation through the production of eicosanoids, especially prostaglandin E_2_ (PGE_2_). PGE_2_ is known to contribute to atherosclerosis and cardiovascular disease. cPLA2 hydrolyses glycerophospholipids into arachidonic acid. Cyclooxygenase (COX) enzymes then convert arachidonic acid into prostaglandins. Non-steroidal anti-inflammatory drugs inhibit COX enzymes. The inhibition of COX enzymes increases myocardial infarction risk [112]. These studies suggest cPLA2 may be involved during myocardial infarction. The contribution of cPLA2 specifically to atherosclerosis has been less studied, but there are a few studies suggesting involvement. Patients with advanced-stage cardiovascular disease had increased vascular cPLA2 expression compared to those with early-stage cardiovascular disease [113]. Treatment with the cPLA2 inhibitor AACOF3 in a cholecalciferol-overload mouse model significantly reduced vascular calcification [113]. These studies suggest cPLA2 is involved in vascular calcification during advanced atherosclerosis. There is also evidence that low-density lipoproteins increase the activity of cPLA2 by participating with secretory PLA2 to increase the release of arachidonic acid in monocytes after inflammatory stimuli [114]. Though limited, these studies do provide evidence that cPLA2 does contribute to atherosclerosis.

## 7. Lipin 1

Lipin-1 is a phosphatidic acid phosphatase that belongs to the evolutionarily conserved family of lipins [115]. Of the three-membered lipin family, lipin-1 exhibits the highest phosphatidate-specific phosphohydrolase activity [116]. Lipin-1 converts PA to DAG via its phosphohydrolase activity in a Mg^2^+-dependent reaction [116,117]. The lipin family has two domains that are conserved from yeast to mammals [117,118]. There are sequence motifs between the N-terminal (N-LIP) and C-terminal (C-LIP) domains that mediate the functions of the lipins [117,119]. Close to the N-LIP is a nuclear localization sequence that translocates lipin-1 to the nucleus [120]. The C-LIP contains the haloacid dehalogenase (HAD)-like phosphatase motif (DXDXT) and an α-helical leucine-rich motif (LXXIL) that mediate the enzymatic and transcriptional co-regulatory activities, respectively [119,121,122]. Three isoforms (lipin1α, lipin1β, and lipin1γ) of lipin-1 are known to be present in humans as a result of the alternative mRNA splicing of the lipin-1 gene [123]. In contrast to in humans, lipin-1γ is not present in mice [122,123]. These splice variants have similar and complementary functions, even though they are differentially expressed in tissues [122,123].

Lipin-1-mediated DAG production is a key step in the biosynthesis of triacylglycerol (TAG), phosphatidylcholine (PC), and phosphatidylethanolamine (PE) [124,125,126]. Lipin-1 resides in the cytosol and can translocate to the endoplasmic reticulum (ER) upon dephosphorylation [127]. Lipin-1 then moves along the membrane to interact with and dephosphorylate PA to generate DAG [128]. Neither the membrane composition nor fatty acid tails of PA influence lipin-1 activity. Lipin-1’s contribution to TAG, PE, and PC production is critical to lipid droplet (LD) generation, which aids in the storage of excess cholesterol, and TAG protects against lipid toxicity [129]. The shRNA depletion of lipin-1 reduced lipid droplet formation in oxLDL-fed RAW264.7 macrophages [125]. The siRNA depletion of lipin-1 in human macrophages reduces LD size and number, and TAG composition in response to fatty acid feeding [130,131]. Additionally, lipins can also protect against dietary glucose toxicity through the regulation of polyunsaturated fatty acid (PUFA) production. In *Caenorhabditis elegans*, lipin prevents dietary glucose toxicity, which leads to a shorter life span [132]. In addition to modulating lipid levels to protect against metabolite overloads, lipin-1 is important in the regulation of autophagy. Autophagy is a housekeeping mechanism of recycling nutrients and degrading dead organelles. Lipin-1-mediated DAG production regulates autophagosome formation and maturation by activating protein kinase D and subsequent VPS34 activity [133]. In support of this, CRISPR-generated lipin-1-deficient myoblasts were observed to have impaired mitochondrial function and irregular autophagic vacuoles under conditions of induced starvation [134]. Thus, lipins and especially lipin-1 are a critical regulatory node in nutrient handling within cells.

The phosphorylation of lipin-1 on multiple sites by mechanistic target of rapamycin complex-1 (mTORC-1) results in retention in the cytosol [135]. Lipin-1 acts as a transcriptional coactivator or repressor by forming a complex with transcription factors such as PPARy, PPARα, and peroxisome proliferator-activated receptor γ coactivator-1α (PGC1α) [119,136,137,138]. PPARs promote macrophage wound-healing activities [139]. Lipin-1 is able to coactivate these transcription factors and enhance their activity. Lipin-1’s transcriptional co-regulatory activity directly facilitates the polarization of IL-4-stimulated macrophages into a wound-healing phenotype [140]. Lipin-1 also acts as a repressor for pro-inflammatory transcription factors such as sterol-response element binding protein-1 (SREBP-1) and nuclear factor of activated T cells isoform c4 (NFATc4) by preventing their binding to promoters [135,141].

Inflammatory responses contribute to the pathogenesis of various diseases. Lipin-1 facilitates the production of eicosanoids by activating cPLA2α to release arachidonic acid from phospholipids [142,143]. Several studies have shown that lipin-1 couples lipid synthesis with pro-inflammatory responses in macrophages [130,144]. Lipin-1 mediates the inflammatory response during TLR4 activation [130]. This process occurs in a diacylglycerol-dependent mechanism that regulates the activation of MAPKs and AP-1 to induce the expression of pro-inflammatory genes [130]. These findings were further supported by an in vivo experiment, which showed that mice lacking lipin-1 experienced earlier weight recovery in response to LPS treatment [130]. The faster recovery observed in lipin-1-deficient mice was due to the reduced expression of pro-inflammatory factors [130]. Lipin-1’s enzymatic activity mediates macrophage pro-inflammatory responses. The uptake of oxLDLs leads to a diacylglycerol-dependent pro-inflammatory signaling cascade that is mediated by lipin-1 [144]. The activation of diacylglycerol-responsive proteins leads to the persistent activation of the pro-inflammatory PKC-MAPK-AP-1 signal transduction pathway [144]. The lipin-1-mediated production of DAG has also been shown to be implicated in colon cancer [145]. DAG increases the expression of pro-inflammatory cytokines in colon-resident macrophages to drive the transformation of dysplastic cells into cancerous cells [145].

In humans, loss-of-function mutations of lipin-1 result in fatal episodic childhood rhabdomyolysis [146,147]. Polymorphisms of LPIN1 are associated with an increased body mass index, type II diabetes, and metabolic syndrome, which are risk factors for atherosclerosis [148]. These results highlight the potential contribution of lipin-1 to cardiovascular disease in humans. In mice, the loss of lipin-1 results in lipodystrophy, although this is not seen in humans, likely due to compensatory mechanisms [149]. Additionally, in mice, lipin-1 contributes to the pathophysiology of fatty liver disease, colon cancer, and atherosclerosis through the promotion of macrophage pro-inflammatory responses [144,145,150]. In addition, lipin-1 was found to colocalize with macrophages in human atherosclerotic plaques [125]. Lipin-1’s enzymatic activity has been implicated in the development of atherosclerosis, as it facilitates the formation of the lipid-laden macrophage phenotype and the production of inflammatory cytokines [144]. Mice lacking myeloid-associated lipin-1 enzymatic activity have a reduction in atherosclerosis [144]. The persistent production of DAG activates a signaling cascade that increases the production and secretion of pro-inflammatory mediators such as IL-6, IL-1, TNF-α, CCL2, and PGE_2_ in response to oxLDL and LPS [125,144]. Lipin-1-deficient macrophages produce significantly less pro-inflammatory cytokines [125]. Collectively, the coupled effect of enhanced modLDL uptake and poor cholesterol efflux lead to the production of tissue-damaging inflammatory mediators that promote atherogenesis and contribute to the different stages of atherosclerosis.

The contributions of macrophage-associated lipin-1 transcriptional co-regulatory activity to atherosclerosis have not yet been published. However, there are data that suggest lipin-1 transcriptional co-regulatory activity may be involved in atherosclerosis. Lipin-1 transcriptional co-regulatory activity increases wound healing and induces macrophage wound-healing/pro-resolving polarization [140]. Macrophage wound-healing responses reduce atherosclerosis plaque growth and severity [151]. Lipin-1 transcriptional co-regulatory activity also augments PPAR promoter binding and increases PPAR-associated genes [137]. PPARs reduce early atherosclerosis progression and enhance atherosclerosis regression [136,139,152,153,154]. Combined, these data suggest that macrophage-associated lipin-1 transcriptional co-regulatory activity would reduce atherosclerosis severity. More work needs to be completed to understand how macrophage-associated lipin-1 transcriptional co-regulatory activity affects atherosclerosis.

## 8. Conclusions

Phospholipids, the components they store, and phospholipases are dynamic regulators of immune cell function. Specifically, the production and removal of bioactive lipids contributes to cellular activation, phagocytosis, ROS generation, cytokine production, and prostanoid production. Phospholipase activity is evident in almost all immune cells. The targeting of the immune system to reduce atherosclerosis is a therapeutic goal that offers a chance to reduce cardiovascular disease. We must define a mechanism of immune responses that can be targeted in atherosclerosis that does not cause global immuno-suppression. Phospholipases may represent one such target. The contribution of phospholipases to atherosclerosis must be further investigated beyond the current understanding. Future work would need to find ways to target phospholipases within the plaque. Numerous small-molecule inhibitors of phospholipase are known, and pairing with nanotechnology may be feasible [155]. The dual function of lipin-1 may also represent an interesting target for atherosclerosis therapeutics. Future work on understanding how lipin-1 is regulated in macrophages, what dictates when each lipin-1 activity will be dominant, and mechanisms to control each lipin-1 activity is needed. The further understanding of the interface of phospholipases, immune cell function, and atherosclerosis will uncover new therapeutic targets and add to our ability to better treat and prevent cardiovascular disease.
